# The Five “Ws” of Frailty Assessment and Chronic Lymphocytic Leukemia: Who, What, Where, Why, and When

**DOI:** 10.3390/cancers15174391

**Published:** 2023-09-02

**Authors:** Isabel González-Gascón-y-Marín, Mónica Ballesteros-Andrés, Sara Martínez-Flores, Ana-E Rodríguez-Vicente, Claudia Pérez-Carretero, Miguel Quijada-Álamo, Alberto Rodríguez-Sánchez, José-Ángel Hernández-Rivas

**Affiliations:** 1 Department of Hematology, University Hospital Infanta Leonor, 28031 Madrid, Spain; 2 Department of Hematology, University General Hospital Gregorio Marañón, 28009 Madrid, Spain; 3 Department of Geriatric Medicine, University Hospital Infanta Leonor, 28031 Madrid, Spain; 4 IBSAL, IBMCC, CSIC, Cancer Research Center, University of Salamanca, 37007 Salamanca, Spain; 5 Department of Hematology, University Hospital of Salamanca, 37007 Salamanca, Spain; 6 Department of Medicine, Complutense University, 28040 Madrid, Spain

**Keywords:** chronic lymphocytic leukemia, comprehensive geriatric assessment, targeted therapies, frailty, comorbidities, age, geriatric domains

## Abstract

**Simple Summary:**

Recent advances in treating elderly patients with chronic lymphocytic leukemia (CLL) have emphasized the importance of geriatric assessment (GA) to evaluate patient fitness and predict treatment outcomes. Targeted therapies (BTK inhibitors and venetoclax) have demonstrated significant clinical benefits and are now a reality in CLL treatment. They have a different toxicity profile that may affect frailty. Therefore, incorporating GA before treatment initiation, considering physical and cognitive function, emotional health, comorbidity, polypharmacy, nutrition, and social support, is essential.

**Abstract:**

Chronic lymphocytic leukemia (CLL) is a disease of the elderly, but chronological age does not accurately discriminate frailty status at the inter-individual level. Frailty describes a person’s overall resilience. Since CLL is a stressful situation, it is relevant to assess the patient´s degree of frailty, especially before starting antineoplastic treatment. We are in the era of targeted therapies, which have helped to control the disease more effectively and avoid the toxicity of chemo (immuno) therapy. However, these drugs are not free of side effects and other aspects arise that should not be neglected, such as interactions, previous comorbidities, or adherence to treatment, since most of these medications are taken continuously. The challenge we face is to balance the risk of toxicity and efficacy in a personalized way and without forgetting that the most frequent cause of death in CLL is related to the disease. For this purpose, comprehensive geriatric assessment (GA) provides us with the opportunity to evaluate multiple domains that may affect tolerance to treatment and that could be improved with appropriate interventions. In this review, we will analyze the state of the art of GA in CLL through the five Ws.

## 1. Introduction

**Figure 1:**
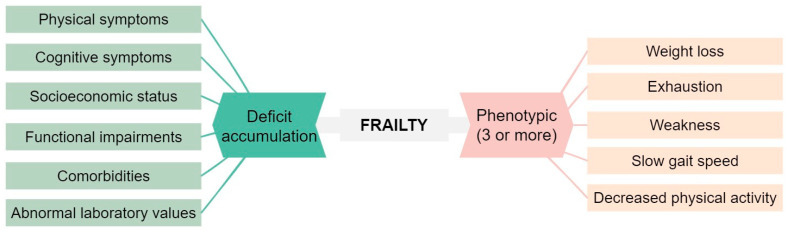
Classical approaches to define frailty [2,3].

Chronic lymphocytic leukemia (CLL) is a prevalent hematological malignancy that predominantly affects older individuals, who are more susceptible to comorbidities and age-related impairments [1]. Comorbidities, age, and frailty are closely related. Frailty is a condition characterized by a decline in physiological reserves and an increased vulnerability to stressors, resulting in a higher risk of adverse outcomes. As depicted in Figure 1 there are two commonly employed approaches for defining frailty [2,3,4].

CLL is considered an indolent disease, and in its early stages, it may remain asymptomatic. Some CLL patients may be under active surveillance, while others with CLL-derived symptoms may require treatment [5]. Currently, targeted therapies have become the cornerstone of CLL treatment, replacing the previous reliance on chemo (immuno) therapy (CIT) [6,7]. These molecules exhibit distinct toxicity profiles compared to conventional CIT. This has resulted in redefining treatment goals due to the favorable tolerability and subsequent stabilization of patients’ quality of life. In addition, factors such as treatment adherence and pharmacological interactions become more relevant [8].

Comprehensive geriatric assessment (GA) is a standardized and reproducible tool used to evaluate the state of frailty beyond clinical observation. Much of the evidence supporting the benefits of GA stems from the era in which CIT was predominant. The International Society of Geriatric Oncology (SIOG) Task Force published a position paper in 2017 providing specific recommendations for managing CLL in elderly patients. At that time, CIT was still an option and only ibrutinib and idelalisib were available. However, since then, venetoclax and other BTK inhibitors (BTKi) have been added to the therapeutic arsenal, rendering some of the previous recommendations outdated [9]. Furthermore, the updated 2023 American Society of Cancer Oncology (ASCO) guidelines on managing vulnerabilities in older patients extend their recommendations beyond chemotherapy to immunotherapy and targeted therapies [10].

The prevalence of frailty in community-dwelling older adults aged 70 tends to be around 15–30%, with differences depending on the studied population, the methodology employed, and the geographical context [11,12]. While age, performance status, and comorbidities have received significant attention in CLL research, the specific evaluation of frailty has not been as extensively explored. In addition, the terms frailty and comorbidity have been used interchangeably, leading to further confusion [13,14]. This review aims to analyze the importance of frailty detection and the management of CLL patients in the era of targeted treatments.

## 2. Who?

If we think of the “typical” portrait of a CLL patient, we imagine a man in his seventies who has been referred to the hematology clinic after a routine blood test to check blood glucose or cholesterol levels [15,16]. However, visualizing a broader picture or even a “short-video” of the patient would be more complicated, as the biological, social, and cultural heterogeneity between individuals is abysmal and gives rise to an infinite number of possibilities. Here is where personalized medicine comes into effect, and we can proudly assert that in many institutions around the world, it is being implemented in the management of CLL patients.

### 2.1. Age and CLL

Similar to other cancers, CLL is an age-related disease with an estimated incidence rate of about 28.7 cases per 100,000 individuals over the age of 65, whereas the rate drops to 8 cases per 100,000 among those aged 50–64 years. Moreover, the prevalence is quadrupled in people over 65, according to data from the United States (164,846 (≥65) vs. 39,113 (50–64)) [15].

Age itself has been demonstrated to be an adverse prognostic marker for CLL despite clinical stage or treatment [17,18,19]. In the preceding era, age was firmly established as an unfavorable predictor [20,21,22,23]. However, the influence of age on prognosis with targeted therapies seems to be less pronounced, at least in clinical trials [24,25,26,27,28].

Elderly patients are often underrepresented in clinical trials involving cancer patients. In contrast, in the context of novel therapies targeting CLL, the median age in most pivotal trials is around 70 years old or more [29,30,31,32,33]. While it may seem reasonable to extrapolate the data from these studies to the general CLL population, the degree of frailty among enrolled participants remains unknown. Furthermore, the majority of studies lack specific information regarding the participation of patients over 80 years old. The results obtained by the German group in six trials evaluating first-line targeted therapies provide significant insights. Out of the 717 patients recruited, only 4.6% who received at least one dose of treatment were over the age of 80 [34].

### 2.2. Biology Disease according to Age

Different retrospective studies have exhibited that the most important biological biomarkers of the disease are the same among young or older patients. Elderly patients present with a more advanced disease at diagnosis according to Rai stage but immunoglobulin heavy-chain (IGHV) mutation status and FISH abnormalities are quite similar [18,19,35] (Table 1).

Therefore, we can speculate that once the disease appears, the biological mechanisms involved in its progression are independent of age. However, *TP53* mutation status information was not available in the studies summarized in Table 1. *TP53* has been linked to tumor development in the elderly in other cancers [36]. Providing further context, a German study with data from 909 newly diagnosed CLL patients found that 12.4% of those older than 70 years old harbored *TP53* mutations vs. 6.4% of those younger than 70 years (*p* = 0.007). Similarly, 17p deletion was also more frequent in elderly patients in contrast to what has been shown in Table 1. The retrospective nature of the studies encompassed in Table 1 could provide insight into these variations. Consequently, we can conclude that the biological biomarkers of the disease do not substantially change in elderly patients, with the exception of *TP53* due to insufficient available information. Therefore, the biological studies to request before starting treatment should be FISH, IGHV, and *TP53* mutations, regardless of the patient’s frailty status and age, in line with the recommendations of international CLL guidelines [5,6,16].

### 2.3. Life Expectancy and Prognostic Models in CLL

The prognosis of CLL is determined by multiple factors, and life expectancy is often overlooked in treatment guidelines [2,4]. Patients of different ages, even with similar biological and social conditions, may have varying life expectancies. Despite significant improvements with CIT, there is still a risk of under-treatment, particularly among frail patients. Studies involving a large number of patients with CLL indicate that the primary cause of death is directly related to CLL, irrespective of comorbidity or age [37,38,39,40,41]. The use of targeted therapies has led to a decrease in mortality in patients requiring treatment. The challenge lies in finding the right balance between reducing toxicities, improving the disease, and maintaining a good quality of life. Therefore, the use of prognostic tools is recommended to guide therapeutic decisions.

**Figure 2:**
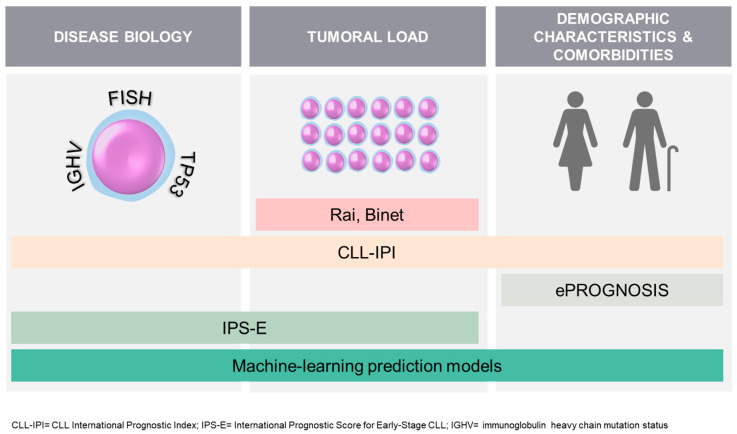
Summary of some tools available to predict the prognosis of patients with CLL at diagnosis.

Binet’s and Rai’s clinical staging systems are still in use today, regardless of recent biological advances [42,43]. To enhance prognostic assessment, the CLL-IPI (CLL International Prognostic Index) was introduced, incorporating the biological factors of the leukemia (*TP53* and IGHV mutation status) along with tumor burden (clinical stage and beta-2-microglobulin levels) and age. It has demonstrated effectiveness in predicting time to first treatment (TTFT) and overall survival (OS) with conventional CIT, as well as in elderly patients with comorbidities [44,45,46,47,48]. However, its usefulness in predicting OS with new treatments is limited [49,50]. More recent is the IPS-E (International Prognostic Score for Early-Stage CLL) which provides a simpler approach by considering only IGHV status and tumor load factors (lymphocyte count, physical examination). It specifically aims to predict TTFT in patients with early Rai/Binet stages and the training cohort in which it was tested included 62.4% of patients aged more than 65 years old [51]. Considerable research and numerous publications have explored alternative prognostic scores for CLL, which have had limited success [52,53,54]. While machine learning algorithms hold promise in improving prognostication, their practical implementation is still pending [55,56]. Lastly, estimating the non-cancer life expectancy of elderly individuals can be carried out using various validated tools listed on ePrognosis [57]. Among these tools are the Lee or Schonberg indexes, recommended in the ASCO guideline for geriatric oncology [10,58]. When estimating non-cancer mortality, using these indexes for CLL patients, it is important to respond “no” to the question regarding the presence of cancer. These tools provide valuable insights but should be considered alongside other factors as part of GA to make well-informed decisions, taking into account their inherent limitations [57]. Figure 2 summarizes these tools. The current challenge is to optimize patient care and outcomes by integrating clinical expertise, patient preferences, and evolving prognostic tools.

## 3. What?

To sharpen the picture and gain clarity on the type of elderly patient with CLL we are dealing with, we must employ GA. This assessment allows us to discern specific details and even provides a clear snapshot of the patient’s movements in the video. Despite the barriers to implementing GA in clinical practice, particularly when there is limited time to evaluate a patient, the mounting clinical evidence strongly supports its effectiveness in patients with hematological malignancies [4,59,60,61,62,63,64,65,66].

### 3.1. Comprehensive Geriatric Assessment

So… what is GA? It is a systematic and multidimensional evaluation of an older adult’s health status, focusing on their medical, functional, cognitive, and psychosocial domains. GA aims to identify the specific needs and vulnerabilities of elderly individuals not captured in routine oncology/hematology care, providing a holistic understanding of their overall health and well-being. This comprehensive approach allows healthcare professionals to develop personalized care plans tailored to the unique requirements of older patients. In recognition of its significance, this concept has been termed “GA guided management (GAM)” in the 2023 ASCO guidelines on managing vulnerabilities in older patients receiving systemic cancer therapy [10].

**Figure 3:**
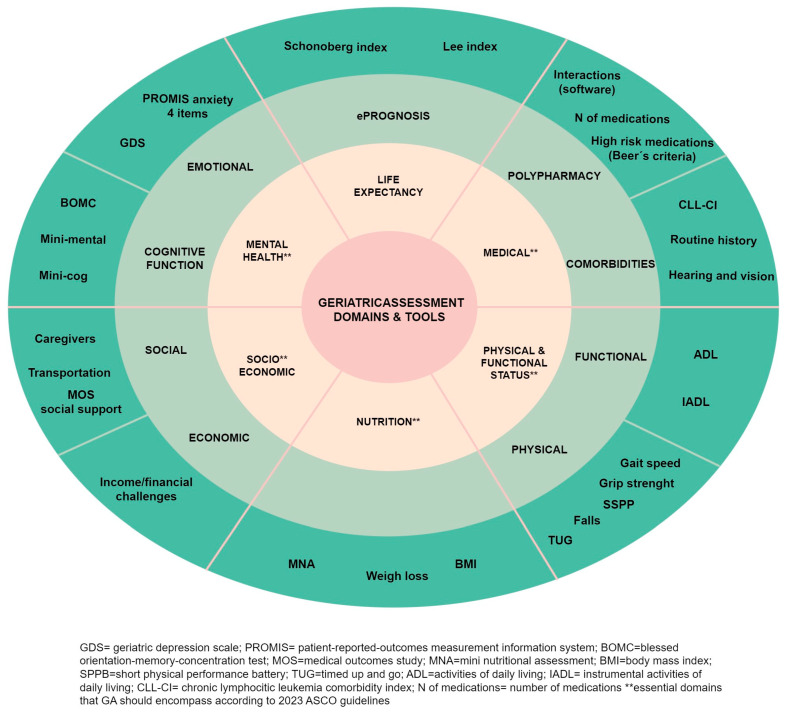
Comprehensive geriatric assessment spheres and tools.

The selection of the specific tool for assessing each domain of GA should be personalized. While we have provided a summary of commonly used options in Figure 3, any validated tool can be used.

The 2017 recommendations from the SIOG for CLL do not indicate a specific preference for a particular tool [9]. Similarly, the 2018 ASCO guidelines on geriatric oncology do not provide the tools to be used, but they do specify the essential assessments that should be conducted [58]. However, these guidelines have been updated recently, in 2023, in light of the results of various clinical trials, which have demonstrated that GAM can improve clinical outcomes [10,67]. In this update, it is recommended that all cancer patients over the age of 65 who are going to receive chemotherapy, as well as immunotherapy or targeted therapies, should undergo GA. This assessment is meant to serve as the foundation for making decisions regarding cancer treatment and should be included in the patient’s individual treatment plan. Additionally, GAM allows for the implementation of targeted interventions based on the assessment results. These interventions may include referrals to physical therapy, supportive care, social work, or nutrition services when appropriate thresholds from validated tests are met. The guideline’s recommendations are clear, evidence-based, and supported by solid evidence. The updated version emphasizes the essential domains that GA should encompass: physical and cognitive function, emotional health, comorbidity, polypharmacy, nutrition, and social support (marked with two asterisks in Figure 3). Additionally, as a novelty, and being aware of the barriers that GA may present (time, resources, and a lack of training on GA), they propose a specific tool named practical geriatric assessment (PGA) as an option. PGA is a patient-reported outcome (PRO) measure that can take between 10 and 25 min and can be completed by the patient or caregiver before the visit or in the waiting area at the office. While PGA has not been validated as a composite tool, the specific items used within it have been strongly validated individually. The PGA tool can be downloaded at https://old-prod.asco.org/sites/new-www.asco.org/files/content-files/practice-patients/documents/2023-PGA-Final.pdf (accessed on 15 August 2023)

Moreover, the guidelines advocate for the incorporation of life expectancy estimation using eprognosis tools and the evaluation of chemotherapy toxicity using CARG (cancer and aging research group) or CRASH (chemotherapy risk assessment scale for high-age patients) tools. These last two instruments are predictive models designated to estimate the risk of chemotherapy toxicity by considering some aspects of GA [68,69]. Nevertheless, these tools may not be applicable to targeted therapies. Notably, a recent study showed that the CARG scale failed to predict toxicity in patients receiving ibrutinib (+/− rituximab) in the phase III ALLIANCE clinical trial (A041202) [70]. Consequently, we have excluded these tools from Figure 3.

Domains such as socioeconomic status and comorbidities may gain increased importance particularly when deciding on finite or continuous therapy in elderly patients. We may come across scenarios where a patient, despite having few comorbidities and a favorable biological profile, is vulnerable due to limited resources for hospital visits. This vulnerability may make him/her a candidate for BTKi, even if initial considerations leaned toward finite therapy with venetoclax. Conversely, another patient with strong family support, a history of severe structural heart disease, anticoagulation, and intestinal angiodysplasia might be offered a venetoclax-based therapy.

The work published by SIOG in 2017, which provided specific recommendations for managing CLL in elderly patients, attempted to categorize patients into three groups based on renal function, comorbidity, and the absence of geriatric deficits: robust, vulnerable, and terminally ill [9]. Given the distinct toxicity profile of targeted therapies and the diminished relevance of renal dysfunction in this context, the current classification may not be suitable.

### 3.2. Comorbidities, Targeted Therapies and CLL

Comorbidities play a pivotal role in the management and prognosis of patients with CLL. The presence of comorbidities can influence treatment selection, dosing, and adherence, as well as contribute to treatment-related toxicities. Consequently, a comprehensive assessment of comorbidities is vital for tailoring personalized treatment plans that balance efficacy and safety in CLL patients receiving targeted therapies. In CLL, the impact of comorbidities on treatment outcomes goes beyond the selection of therapy. Comorbid conditions may also influence OS, treatment response, and the risk of adverse events during targeted therapy [23,39,71].

#### 3.2.1. Scales to Assess Comorbidities

Various general tools are available for assessing a patient’s comorbidities in CLL, such as the Charlson Comorbidity Index (CCI), the National Cancer Institute (NCI), or the Cumulative Illness Rating Scale (CIRS). Among these, the CIRS has been commonly used in clinical trials [31,72]. Recently, the CLL comorbidity index (CLL-CI) was developed to gain a better understanding of the specific impact of comorbidities in CLL [73]. By evaluating vascular, endocrine, and gastrointestinal deficits, patients can be stratified into three risk groups. This index has proven to be a valuable tool for predicting outcomes with CIT and ibrutinib [73,74]. Wider validation across different targeted therapies could further enhance its utility in clinical practice.

#### 3.2.2. Comorbidities with Special Interest with Targeted Therapies

- Cardiovascular comorbidity

The principal class of side effects associated with BTKi is related to the cardiovascular system, with atrial fibrillation (AF) and hypertension being particularly notable due to their frequency, and ventricular arrhythmias due to their severity. As AF and hypertension are relatively prevalent in the elderly population, it is not uncommon for CLL patients to have one of these conditions. Hence, it is crucial to monitor blood pressure and pulse in these patients before starting BTKi and during the course of therapy. Recently, European guidelines on cardio-oncology have been published, offering specific recommendations for BTKi candidates, including the need for a comprehensive baseline cardiac assessment [75,76].

- High bleeding risk or patients under antiaggregant/anticoagulant therapies

Another relevant side effect associated with BTKi is the bleeding risk. Mild bleeding events are relatively common, while moderate to severe bleeding is exceptional. However, in patients with CLL who are already on antiplatelet or anticoagulant therapy for other reasons, the bleeding risk may increase. Concurrent treatment with BTKi and vitamin K antagonist anticoagulants or dual antiplatelet therapy should be avoided, and in such cases, healthcare providers may need to adjust or modify the patient’s medications to minimize the risk of bleeding complications [77].

- Infection

Infections are a frequent concern among patients with CLL, a disorder with severe immune dysfunction. This vulnerability extends to patients undergoing targeted therapies as well. To address this, it is advisable to follow local vaccination schedules and immunize these patients before treatment if they have not received the recommended vaccines (influenza, pneumococcus, COVID-19, varicella-zoster, etc.) Additionally, a personalized assessment should be conducted to determine the need for further prophylaxis against specific infections, such as *Pneumocystis jirovecii* or herpes varicella zoster infections or even intravenous immunoglobulin. By implementing these preventive measures, we can effectively reduce the risk of infections and enhance the overall care and well-being of patients throughout their treatment journey [77,78].

- Renal impairment

Renal insufficiency is another significant comorbidity in CLL. Historically, this was a concern due to the use of fludarabine. Currently, the main risk for patients with renal impairment is the possibility of experiencing tumor lysis syndrome when starting venetoclax, which could lead to worsening renal function. Considerable efforts have been made to mitigate this risk, and with the current guidelines involving dose escalation, hydration, the use of hypouricemic agents or rasburicase, or a debulking phase with anti-CD20 antibodies (CLL14 trial) or BTKi (GLOW trial), this comorbidity is rarely a major concern [79].

- Other comorbidities

BTKi also gives rise to other less severe but more frequent adverse events such as arthralgias, diarrhea, cutaneous toxicity, or headaches. Generally, the patient’s underlying comorbidities do not influence these secondary effects. On the other hand, phosphatidylinositol-3 kinase delta inhibitors (PI3Ki) are not used in CLL due to their diminished effectiveness and high toxicity, particularly immune-related toxicities like colitis, pneumonitis, or hepatotoxicity.

## 4. Where?

GA can be conducted in various healthcare settings, including primary care, hospitals, and specialized geriatric or oncology clinics. Furthermore, there are different modalities for conducting GA, which can be categorized based on different parameters.

Firstly, GA can be conducted through screening, where only vulnerable patients are targeted, or as a full GA without preselection. The screening tools enable the identification of high-risk individuals and can potentially prevent 20–40% of cancer patients from requiring GA. However, in the case of CLL specifically, this percentage may be lower due to the older age and the presence of comorbidities at diagnosis. Notably, these tools have shown their ability to predict mortality in cancer patients [4,58]. Various screening tools are available, such as G8 and Vulnerable Elders Survey-13 (VES13). One particular tool, the GAH brief Geriatric Assessment in Hematology, has been specifically designed for hematological malignancies [80,81,82]. However, the preferred screening tools endorsed by the ASCO and SIOG are the G8 and VES.

**Figure 4:**
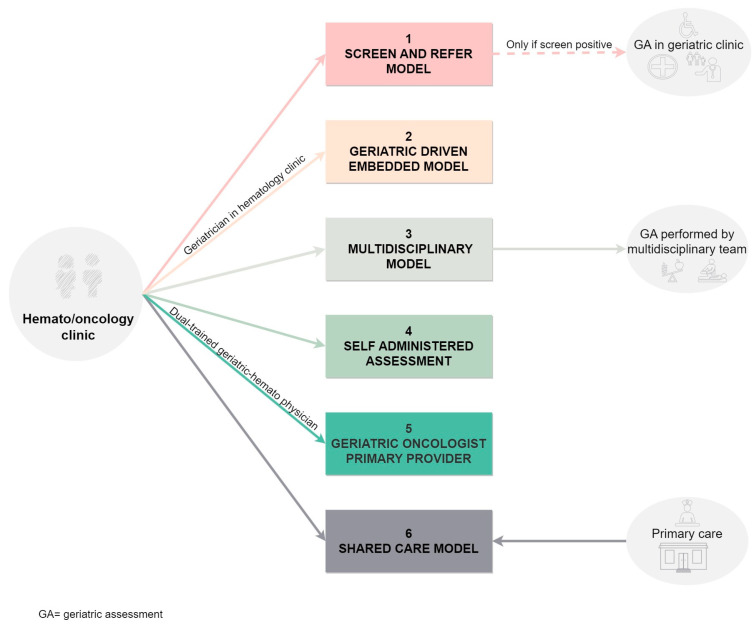
Models of comprehensive geriatric assessment based on the location and the involved health giver.

Secondly, the modalities can be classified considering specialists’ involvement and evaluation locations. This classification, as presented by Chapman and colleagues [83], has been further illustrated in Figure 4.

Thirdly, modalities can be differentiated based on the mode of care, whether it is in person or remote (via telephone or video consultations). The availability of telemedicine and digital healthcare options has made remote GA more accessible, providing greater convenience and flexibility for elderly patients, especially those with reduced mobility [84]. However, it is important to note that telemedicine cannot fully replace the importance of in-person visits. Thus, it should be considered as a supplementary option rather than a complete substitute for traditional in-person medical consultations.

Fourthly, we can examine who performs the GA. It can be partially conducted by the elderly patients themselves or their caregivers through PROMs. Alternatively, healthcare providers can administer the assessment, or it can be a collaborative effort involving both patients and healthcare professionals. Each approach has its unique benefits and challenges, and the choice depends on factors such as patient preference, the complexity of the assessment, and available resources.

No single modality fits all scenarios perfectly. The optimal approach should be adaptable and tailored to the available resources. Combining multiple approaches and incorporating innovative technologies can enhance the effectiveness and efficiency of GA [58,85,86].

## 5. Why?

Having reached this stage, our aim is to have persuaded readers about the ways in which GA can improve the clarity and focus in evaluating elderly CLL patients. Let us now dive into why GA is important in the field of oncology. GA has demonstrated to predict survival outcomes [87], facilitate the detection of treatment-related toxicities [4,68], support decision-making processes [88], provide valuable information on healthcare resource utilization [89], and identify limitations as well as implement interventions such as rehabilitation programs, nutrition supplementation, or care services [90]. However, there is limited evidence supporting the value of GA in hematologic malignancies, with most studies focusing on acute myeloid leukemia and multiple myeloma [4]. Similarly, while the SIOG recommends GA for CLL patients, there is very little evidence to support this recommendation, especially in the context of targeted therapies [9]. We will now summarize the findings from the four studies that establish a correlation between GA and clinical outcomes in CLL (Table 2).

Goede et al. analyzed the results of a multidimensional GA conducted on 75 out of 97 patients enrolled in a clinical trial with fludarabine treatment. Statistically significant correlations were found between impaired cognitive function and event-free survival and between functional and cognitive impairments and OS. The main causes of death in patients with these impaired domains were CLL and deaths of unknown cause. Treatment-related or comorbidity-related mortality was not higher in this group compared to patients without these impairments. The study’s limitations include being conducted on a selected patient cohort; The fact that the GA as not mandatory and did not encompass all domains, with social status and mood being absent; and that there were time-related challenges in conducting the GA. Notably, unlike other studies, comorbidity measured by CIRS did not impact OS, nor was it associated with toxicity [48].

Molica et al. examined a cohort of 108 real-world CLL patients to assess the prognostic impact of a simplified geriatric assessment, including age, comorbidities (CIRS), and physical function. Most patients received chlorambucil (Chl)-based treatments (56.8%), with only six (10.3%) being treated with ibrutinib. Using these variables, they developed a frailty score that categorized patients into three groups: frail, pre-frail, and fit. Moreover, this score successfully predicted OS and TTFT, irrespective of Rai stage. However, the authors question the applicability of this score to the broader CLL population, as most treated patients received Chl, an outdated treatment by today’s standards [92].

The Alliance (A041202) clinical trial is among the few studies that include GA and targeted therapies [28]. The GA was optional, thus out of the 524 participants in the study, 369 consented and underwent the initial GA. Specific domains, rather than complete GA, were able to predict drug discontinuation, progression-free survival (PFS), and OS. The social domain had an impact on PFS and OS after adjusting for baseline characteristics and treatment arms. Nutritional status also influenced PFS. Protective domains against drug discontinuation were low depression and anxiety levels, high social activity scores, and low weight loss in the previous six months. However, none of the domains were associated with toxicity. Interestingly, the CARG calculator did not predict toxicity for any arm of the study. Frail patients in the ibrutinib arms showed a trend toward improvement in each geriatric domain over time. The study’s limitations include the inclusion of predominantly fit patients, the optional nature of the GA, the underrepresentation of certain domains such as cognition, and the limited number of predictors included in the multivariable analysis [70].

Lastly, Van der Straten et al. examined the influence of various geriatric domains on health-related quality of life (HRQoL) and outcomes. In the HOVON 139/GiVe trial, a multicenter, randomized phase II study, 131 previously untreated CLL patients considered unfit for fludarabine-based treatment but with an Eastern Cooperative Oncology Group (ECOG) performance status of 0 or 1 were enrolled. They received fixed-duration treatment with obinutuzumab and venetoclax (Ven-O) and underwent GA and HRQoL evaluations. At baseline, the median number of geriatric impairments was two, with gait speed, nutrition, and comorbidities being the most frequently affected. Patients with ≥2 geriatric impairments had a higher incidence of grade ≥3 non-hematological toxicities. Over time, the number of geriatric impairments decreased and HRQoL showed clinical improvements in various subscales (global health status, physical and emotional functioning, and fatigue). The study suggests that Ven-O improves the well-being of older, unfit CLL patients, regardless of geriatric impairments at baseline. However, the results may have limited generalizability due to the exclusion of patients with ECOG scores of 3–4 [93].

The scarcity of studies and the heterogeneity of therapeutic approaches make it challenging to draw definitive conclusions. Each study has highlighted different aspects of geriatric health as significant. However, with targeted therapies, social and nutritional domains and comorbidities appear to hold particular importance, and interventions in these areas can be beneficial. This underscores the importance of detecting and addressing these factors during patient follow-up to implement necessary modifications for improved outcomes.

## 6. When?

GA must be assessed at least prior to cancer treatment as it may alter planned oncological treatment or result in additional geriatric interventions [4].

As stated before, the treatment of CLL has evolved significantly, from monotherapy with Chl +/− prednisone or fludarabine (F) to the combination of F with cyclophosphamide (C). A major change with impact on OS emerged with the German CLL Study Group’s (GCLLSG8) study [94]. This study compared FC vs. FC-rituximab (FCR), demonstrating an unprecedented superiority in achieving complete responses (CR), PFS, and OS with the addition of rituximab. As a result, FCR became the gold standard in CLL treatment. However, the FCR regimen was not suitable for elderly patients due to toxicity. Indeed, a subsequent study by the same group (GCLLSG10) showed no differences in PFS for FCR compared to bendamustine-rituximab (BR) in patients older than 65 years. Both regimens appeared to be equally effective in this age group, but FCR was more toxic [95]. Consequently, BR became the new recommended standard treatment for patients over 65 years without comorbidities.

The first clinical trial that included unfit patients was the (GCLLSG11) comparing Chl vs. Chl-R vs. Chl-obinutuzumab (Chl-O). Patients were categorized as unfit based on a CIRS score > 6 or a creatinine clearance between 30 and 69 mL/min. This trial demonstrated the superiority of the Chl-O regimen (PFS 26.7 months), consolidating it as the gold standard for unfit patients [96]. Since then, all guidelines have adopted patient categorization based on their frailty status (fit/unfit) using the presence of comorbidities and/or renal failure, instead of relying on a GA.

The first targeted treatments, ibrutinib (BTKi) and idelalisib (PI3Ki), showed efficacy in patients with *TP53* abnormalities, where CIT was very poor. Subsequently, its use became widespread in the relapsed/refractory setting and eventually found its place in first-line treatment. Ibrutinib has the distinction of being the drug with the longest-standing history of success, proving its efficacy regardless of the mutational status of IGHV. Recently the updated results from the RESONATE-2 trial (ibrutinib vs. Chl in patients >65 years without 17p deletion (del17p)) have been published. With a median follow-up of 8 years, ibrutinib maintained a significant PFS benefit over Chl (hazard ratio (HR) 0.154; 95% confidence interval (CI), 0.108–0.220). At 7 years, the PFS rate with ibrutinib was 59% vs. 9% with Chl, and the OS with ibrutinib was 78%. Adverse events of clinical interest were hypertension (20%/year), AF (7–9%/year), hemorrhage, and fatal cardiac events (only 3%) [97].

Given the favorable outcomes with ibrutinib in the frontline setting, the ALLIANCE study (A041202) compared ibrutinib, ibrutinib-R, and BR. The 4-year PFS estimates were 47% (BR) and 76% (ibrutinib and ibrutinib-R), with no differences between the ibrutinib-containing arms. This PFS benefit was observed across all subgroups and was most pronounced in patients with *TP53* abnormalities [98].

As previously mentioned, after the CLL11 trial, Chl-O became the standard treatment for unfit patients at that time, and it was used as the comparator arm in trials. Therefore, ibrutinib-O was compared to Chl-O in the ILLUMINATE trial. After a median follow-up of 45 months, the estimated PFS continued to be significantly longer in the ibrutinib-O arm (not reached vs. 22 months) [72].

The ELEVATE TN trial compared acalabrutinib (a more selective BTKi developed to improve toxicity) +/− O to Chl-O. With a median follow-up of 47.9 months, PFS was not reached for any of the acalabrutinib arms vs. 27.8 months for Chl-O. The benefits were consistent irrespective of IGHV mutation status or *TP53* abnormalities. The most common adverse events were diarrhea, headache, and neutropenia for the acalabrutinib arms. Among adverse events of clinical interest, cumulative incidences of AF and hypertension over time were low [99]. 

The CLL14 clinical trial compared venetoclax-O, the first fixed-duration regimen, with Chl-O. After a median follow-up of 64.5 months, PFS was significantly higher in the Venetoclax-O arm (62.6% vs. 27%). In both arms, patients with *TP53* abnormalities and unmutated IGHV had a longer PFS if treated with Ven-O. However, those with *TP53* abnormalities and unmutated IGHV had a worse prognosis compared to patients without these alterations [31].

Interestingly, the third BTKi, zanubrutinib (developed to improve binding to BTK), was compared with BR in the SEQUOIA clinical trial. Patients without del17p were randomly assigned to zanubrutinib or BR. At a median follow-up of 26.2 months, PFS was significantly improved for patients treated with zanubrutinib (HR 0.42 [95% CI 0.28–0.63]; two-sided *p* < 0·0001) [33].

Ibrutinib and acalabrutinib have been compared in the ELEVATE RR clinical trial. After a median follow-up of 40.9 months, acalabrutinib was determined to be noninferior to ibrutinib with a median PFS of 38.4 months in both arms. All-grade AF/atrial flutter incidence was significantly lower with acalabrutinib vs. ibrutinib (9.4% vs. 16.0%) [100]. Ibrutinib and zanubrutinib have also been compared in the ALPINE clinical trial, which demonstrated the superiority of zanubrutinib over ibrutinib (HR for disease progression or death, 0.65; 95% (CI), 0.49 to 0.86; *p* = 0.002), along with a lower incidence of AF [101].

Lastly, the combination of ibrutinib and venetoclax has also been tested, showing synergistic effects. The GLOW clinical trial compared ibrutinib-venetoclax with Chl-O in unfit CLL patients. Once again, frailty was defined only by comorbidities and renal impairment. With a median follow-up of 27.7 months, PFS was superior for the ibrutinib–venetoclax arm (HR, 0.216; 95% CI, 0.131–0.357; *p* < 0.001) [32]. Seven fatal adverse events resulting in death were reported in the ibrutinib–venetoclax arm, indicating at the beginning of therapy a worse safety profile of the combination in the elderly population. 

Currently, treatment with the new targeted therapies is differentiated into treatments of indefinite or fixed duration. At present, both approaches, along with their combination, are being tested in the frontline setting in a new study led by the German group (GCLLSG17, NCT04608318).

Real-world evidence may aid in understanding the use of targeted treatments outside of clinical trials. Several studies with ibrutinib have highlighted that comorbidity, rather than age, is an adverse factor. There is also some evidence with venetoclax where comparable responses to clinical trial results have been replicated. However, it is worth noting that the median age of patients in these real-world studies tends to be lower than 71 years, which may not fully reflect the actual clinical practice [71,102,103,104].

**Figure 5:**
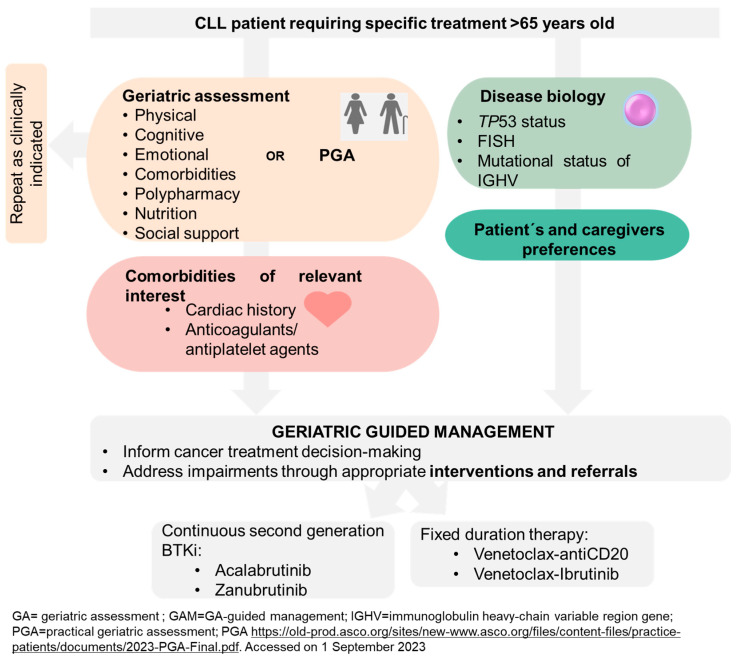
Proposed treatment algorithm based on geriatric assessment for elderly cancer patients.

The distinct toxicity profiles of these novel agents, different from traditional CIT, and their assessment in unfit patients have obviated the need to categorize patients as fit or unfit in current guidelines [7,105,106]. We critique this decision based on the evidence presented throughout this review. A CIRS > 6 implies comorbidity, one of the spheres assessed in GA, and a creatinine clearance < 70 limits the use of fludarabine, a drug already abandoned in CLL treatment. The alteration of other spheres, such as cognition, functional status, nutrition, and social support, has been shown to impact at least the PFS in these patients [70,92]. We believe that interventions in many of these domains can be made to influence the quality of life of our patients. Does it make sense to administer highly effective treatments, with apparently acceptable toxicities, to patients whose quality of life is being impacted by not intervening in their frailty? Therefore, we propose a treatment algorithm that takes all these variables into account, as depicted in Figure 5. This algorithm serves as a comprehensive guide for clinicians to tailor cancer treatment plans based on individual GA results and specific patient needs. By incorporating these considerations, we aim to optimize treatment outcomes and enhance the overall well-being of elderly cancer patients.

Pending further evidence, we consider it appropriate to perform a geriatric evaluation or, alternatively, screening of all patients > 65 years of age before treatment. CLL patients continue to age and develop frailty; therefore, their frailty should be reassessed as clinically indicated (for example after 3–12 months), particularly in continuous treatments. Further research is required to evaluate the effect of the side effects of targeted treatments and geriatric interventions on frailty, outcomes, and quality of life. In addition, evidence from a narrative review suggests potential positive cost-effectiveness, particularly in reducing hospital stay and treatment toxicity, supporting GA integration into geriatric oncology practice [107].

## 7. Conclusions

In this review, we embarked on a journey to explore the five Ws of frailty assessment in CLL, seeking to shed light on the intricacies of personalized care in the age of targeted therapies. Just like a snapshot captures a single moment, understanding frailty in CLL demands a GA. This approach shines a light on the unique vulnerabilities and needs of elderly individuals, often overlooked in routine oncology care, enabling tailored care plans.

As we progress through the evolving landscape of CLL management, a new picture emerges, revealing the distinct toxicity profiles of targeted therapies and the challenge of assessing frailty. A diverse patient population demands a precise and focused lens to achieve optimal outcomes. While the biology of the disease remains crucial, the evidence we have presented suggests that it should no longer be the sole determinant in deciding the type of treatment. Quality of life and interventions play pivotal roles and further research must be dedicated to these aspects.

However, implementing GA can be challenging due to limited time, resources, and a lack of trained personnel. Yet, potential solutions such as the use of PROMs or the recently proposed practical geriatric assessment by ASCO may offer a practical tool that is easy to implement, even in limited-resource settings.

The lens of frailty assessment helps us move away from categorizing patients as unfit only based on comorbidities and renal function. Instead, we harness the power of GA to obtain a more accurate picture of each patient’s needs and implement targeted interventions. Guided by the light of frailty assessment, we hope to be able to paint a brighter future for every CLL patient.

## Figures and Tables

**Table 1:** Differences in patient and biological characteristics according to age in CLL in 3 different studies from different countries.

	Spanish Cohort [18]				*p*	Chinese Cohort [35]		*p*	Mayo Clinic Cohort [19]				*p*
N	949					601			2487				
Years since diagnosis	1990–2012					2010–2021			1995–2008				
Age	<59	60–69	70–79	>80		<60	≥60		<55	55–64	65–74	≥75	
Sex, male (%)	65	59	51	53	0.004	55.4	55.2	NS	68	69	66	68	NS
Rai (%)													
0	48	66	62	63	<0.001	7.5	7.6	NS	43	56	57	58	<0.01
I/II	47	29	29	19		51.3	48.3		53	38	38	31	
III/IV	5	5	9	18		41.2	44.2		4	6	6	12	
IGHV unmutated (%)	51	39	44	43	NS	34.3	32.6	NS	49	46	41	41	NS
FISH (%)													
del 13q	32	38	39	42	NS	39.3	36.3	NS	44	43	41	34	NA
Normal	27	33	29	30		32.1	42.9		27	24	26	28	
+12	14	13	15	9		14.3	15.4		16	20	19	23	
del 11q	15	10	10	12		19.6	9.9		8	8	7	11	
del 17p	12	6	7	7		12.5	14.3		4	3	5	4	

N = number; del 13q = deletion 13q; +12 = trisomy 12; del 11q = deletion 11q; del 17p = deletion 17p; NS = non statistically significant; NA = not available.

**Table 2:** Principal characteristics and results of the studies that used GA in CLL patients.

	Goede et al. 2016 [91]	Molica et al. 2019 [92]	Johnson et al. 2023 [70]	Van der Straten et al. 2023 [93]
Study type	Phase 3 clinical trial (CLL9)	Real life	Phase 3 clinical trial (ALLIANCE)	Phase 2 trial (HOVON 139/GiVe)
Treatments and N of patients	Fludarabine +/− darbopoetin α (N = 97)	No treatment (50)	BR (N = 113)	Ven-O (N = 67)
		Treatment (58)	Ibrutinib (N = 130)	
		Chl (56.8%)	Ibrutinib-R (N = 126)	
		Chl-R (8.6%)		
		BR (18.9%)		
		Ibrutinib (10.3%)		
		FCR (5.1%)		
Baseline characteristics				
Age of patients	75 (range, 48–87)	71 (range, 65–90) 32.7% > 75 years old	71 (range 65–87)	71 (IQR 68–75) 69% > 79 years old
Comorbidities	Median CIRS 5 (0–23); CIRS > 6 36%	CIRS > 6 15.7%	Median N 2 (range 0–14)	CIRS 3 (1–5), CIRS ≥ 7 20%
ECOG (median (range)	1 (0–2)	0–4 included but % NA	0–1 --> 98%; 2 --> 2%	Only 0–1
GA domains				
Comorbidities	CIRS	CIRS > 6 15.7%	N of comorbidities	CCI
Functional	IADL, timed “Up & Go”	ADL, IADL	ADL, timed “Up & Go”, MOS-physical functioning score	ADL, IADL, grip strength
Mood			Anxiety score, mood score	GDS
Nutrition	MNA		Unintentional weight loss in last 6 months, BMI	MNA
Cognition	DEMTECT		BOMC	MMSE
Social			MOS-SSS, social scores	
Geriatric syndromes			Falls	Sarcopenia EWGSOP1 and 2, muscle mass (SMI) and quality (MRA)
Toxicity scale			CARG	
HRQoL				EORTC-QLC &C30 and CLL17
Moment of GA	Prior to treatment initiation	NA	Prior to therapy	Prior to therapy
			After 6 months	After 12 months
			After 2 years or at discontinuation	After 15 months
Median follow-up time	NA	66 months (range, 3–330)	NA	44 months
Most common impairments	Comorbidities	NA	Social, nutritional, functional	Comorbidities, gait speed, and nutrition
Association of GA domains and outcomes				Analyzed N of impairments. Did not analyze each geriatric domain
Toxicity	Treatment delays associated with functional impairment		None	Toxicity if ≥2 impairments
PFS	Cognition		Social support, nutritional	No impact
OS	Functional and cognition	Age (>75), CIRS > 4, ADL < 5	Social support	No impact
Evolution over time	NA	NA	Improvements in performance status and weight; reduction in anxiety	Improvement of HRQoL over time irrespective of N of geriatric impairments
Others		Made a frailty score that divided patients into 3 groups (fit, intermediate-fit, and frail) able to predict OS	CARG did not predict toxicity	

GA = comprehensive geriatric assessment; HRQoL = health-related quality of life; PFS = progression-free survival; OS = overall survival; CLL = chronic lymphocytic leukemia; N = number; CIRS = cumulative illness rating scale; CCI = Charson comorbidity index; IADL = instrumental activities of daily living; MNA = mini nutritional assessment; DEMTEC = Dementia Toolkit for Effective Communication; NA = not available; R = rituximab; Chl = chlorambucil; B = bendamustine; FCR = fludarabine, cyclophosphamide, rituximab; ADL = activities of daily living; CARG = cancer aging research group; BOMC = Blessed Orientation–Memory–Concentration Test; MOS = medical outcomes study; SSS = social support survey; IQR = interquartile range; GDS = global deterioration scale; MMSE = mini-mental state; EWGSOP = European workgroup for sarcopenia; SMI = skeletal muscle mass index; MRA = muscle radiation attenuation.

## Data Availability

The data presented in this study are available in this article.

## References

[1] Balducci L., Dolan D. (2015). Chronic Lymphocytic Leukemia in the Elderly: Epidemiology and Proposed Patient-Related Approach. Cancer Control.

[2] Fried L.P., Tangen C.M., Walston J., Newman A.B., Hirsch C., Gottdiener J., Seeman T., Tracy R., Kop W.J., Burke G. (2001). Frailty in older adults: Evidence for a phenotype. J. Gerontol. A Biol. Sci. Med. Sci..

[3] Rockwood K., Song X., MacKnight C., Bergman H., Hogan D.B., McDowell I., Mitnitski A. (2005). A global clinical measure of fitness and frailty in elderly people. CMAJ.

[4] Scheepers E.R.M., Vondeling A.M., Thielen N., van der Griend R., Stauder R., Hamaker M.E. (2020). Geriatric assessment in older patients with a hematologic malignancy: A systematic review. Haematologica.

[5] Kay N.E., Hampel P.J., Van Dyke D.L., Parikh S.A. (2022). CLL update 2022: A continuing evolution in care. Blood Rev..

[6] Eichhorst B., Robak T., Montserrat E., Ghia P., Niemann C.U., Kater A.P., Gregor M., Cymbalista F., Buske C., Hillmen P. (2021). Chronic lymphocytic leukaemia: ESMO Clinical Practice Guidelines for diagnosis, treatment and follow-up. Ann. Oncol..

[7] CLL/SLL NCCN Guidelines. https://www.nccn.org/professionals/physician_gls/pdf/cll.pdf.

[8] Rhodes J.M., Barrientos J.C., Rai K.R. (2022). How Have Targeted Agents Changed the Treatment Landscape for Elderly Patients with CLL?. Curr. Oncol. Rep..

[9] Stauder R., Eichhorst B., Hamaker M.E., Kaplanov K., Morrison V.A., Österborg A., Poddubnaya I., Woyach J.A., Shanafelt T., Smolej L. (2017). Management of chronic lymphocytic leukemia (CLL) in the elderly: A position paper from an international Society of Geriatric Oncology (SIOG) Task Force. Ann. Oncol..

[10] Dale W., Klepin H.D., Williams G.R., Alibhai S.M.H., Bergerot C., Brintzenhofeszoc K., Hopkins J.O., Jhawer M.P., Katheria V., Loh K.P. (2023). Practical Assessment and Management of Vulnerabilities in Older Patients Receiving Systemic Cancer Therapy: ASCO Guideline Update. J. Clin. Oncol..

[11] Ofori-Asenso R., Chin K.L., Mazidi M., Zomer E., Ilomaki J., Zullo A.R., Gasevic D., Ademi Z., Korhonen M.J., LoGiudice D. (2019). Global Incidence of Frailty and Prefrailty Among Community-Dwelling Older Adults: A Systematic Review and Meta-analysis. JAMA Netw. Open.

[12] Rivas-Ruiz F., Machón M., Contreras-Fernández E., Vrotsou K., Padilla-Ruiz M., Díez Ruiz A.I., de Mesa Berenguer Y., Vergara I. (2019). Group GIFEA Prevalence of frailty among community-dwelling elderly persons in Spain and factors associated with it. Eur. J. Gen. Pr..

[13] Frustaci A.M., Deodato M., Zamprogna G., Cairoli R., Montillo M., Tedeschi A. (2022). SOHO State of the Art Updates and Next Questions: What is Fitness in the Era of Targeted Agents?. Clin. Lymphoma Myeloma Leuk..

[14] Tedeschi A. (2020). What is Fitness in the Era of Targeted Agents?. Clin. Lymphoma Myeloma Leuk..

[15] SEER*Explorer Application. https://seer.cancer.gov/statistics-network/explorer/application.html?site=93&data_type=1&graph_type=2&compareBy=sex&chk_sex_3=3&chk_sex_2=2&hdn_rate_type=1&race=1&age_range=1&hdn_stage=101&advopt_precision=1&advopt_show_ci=on&hdn_view=0&advopt_show_apc=on&advopt_display=2#resultsRegion0.

[16] Hallek M., Cheson B.D., Catovsky D., Caligaris-Cappio F., Dighiero G., Döhner H., Hillmen P., Keating M., Montserrat E., Chiorazzi N. (2018). iwCLL guidelines for diagnosis, indications for treatment, response assessment, and supportive management of CLL. Blood.

[17] (2016). International CLL-IPI working group An international prognostic index for patients with chronic lymphocytic leukaemia (CLL-IPI): A meta-analysis of individual patient data. Lancet Oncol..

[18] Baumann T., Delgado J., Santacruz R., Martínez-Trillos A., Royo C., Navarro A., Pinyol M., Rozman M., Pereira A., Villamor N. (2014). Chronic lymphocytic leukemia in the elderly: Clinico-biological features, outcomes, and proposal of a prognostic model. Haematologica.

[19] Shanafelt T.D., Rabe K.G., Kay N.E., Zent C.S., Jelinek D.F., Reinalda M.S., Schwager S.M., Bowen D.A., Slager S.L., Hanson C.A. (2010). Age at diagnosis and the utility of prognostic testing in patients with chronic lymphocytic leukemia. Cancer.

[20] Strugov V., Stadnik E., Virts Y., Andreeva T., Zaritskey A. (2018). Impact of age and comorbidities on the efficacy of FC and FCR regimens in chronic lymphocytic leukemia. Ann. Hematol..

[21] Vojdeman F.J., Van’t Veer M.B., Tjønnfjord G.E., Itälä-Remes M., Kimby E., Polliack A., Wu K.L., Doorduijn J.K., Alemayehu W.G., Wittebol S. (2017). The HOVON68 CLL trial revisited: Performance status and comorbidity affect survival in elderly patients with chronic lymphocytic leukemia. Leuk. Lymphoma.

[22] Satram-Hoang S., Reyes C., Hoang K.Q., Momin F., Skettino S. (2014). Treatment practice in the elderly patient with chronic lymphocytic leukemia-analysis of the combined SEER and Medicare database. Ann. Hematol..

[23] Goede V., Cramer P., Busch R., Bergmann M., Stauch M., Hopfinger G., Stilgenbauer S., Döhner H., Westermann A., Wendtner C.M. (2014). Interactions between comorbidity and treatment of chronic lymphocytic leukemia: Results of German Chronic Lymphocytic Leukemia Study Group trials. Haematologica.

[24] Fresa A., Autore F., Galli E., Tomasso A., Stirparo L., Innocenti I., Laurenti L. (2021). Treatment Options for Elderly/Unfit Patients with Chronic Lymphocytic Leukemia in the Era of Targeted Drugs: A Comprehensive Review. J. Clin. Med..

[25] Fischer K., Al-Sawaf O., Fink A.-M., Dixon M., Bahlo J., Warburton S., Kipps T.J., Weinkove R., Robinson S., Seiler T. (2017). Venetoclax and obinutuzumab in chronic lymphocytic leukemia. Blood.

[26] Burger J.A., Tedeschi A., Barr P.M., Robak T., Owen C., Ghia P., Bairey O., Hillmen P., Bartlett N.L., Li J. (2015). Ibrutinib as Initial Therapy for Patients with Chronic Lymphocytic Leukemia. N. Engl. J. Med..

[27] Ghia P., Pluta A., Wach M., Lysak D., Kozak T., Simkovic M., Kaplan P., Kraychok I., Illes A., de la Serna J. (2020). ASCEND: Phase III, Randomized Trial of Acalabrutinib Versus Idelalisib Plus Rituximab or Bendamustine Plus Rituximab in Relapsed or Refractory Chronic Lymphocytic Leukemia. J. Clin. Oncol..

[28] Woyach J.A., Ruppert A.S., Heerema N.A., Zhao W., Booth A.M., Ding W., Bartlett N.L., Brander D.M., Barr P.M., Rogers K.A. (2018). Ibrutinib Regimens versus Chemoimmunotherapy in Older Patients with Untreated CLL. N. Engl. J. Med..

[29] Burger J.A., Barr P.M., Robak T., Owen C., Ghia P., Tedeschi A., Bairey O., Hillmen P., Coutre S.E., Devereux S. (2020). Long-term efficacy and safety of first-line ibrutinib treatment for patients with CLL/SLL: 5 years of follow-up from the phase 3 RESONATE-2 study. Leukemia.

[30] Isaac K., Mato A.R. (2020). Acalabrutinib and Its Therapeutic Potential in the Treatment of Chronic Lymphocytic Leukemia: A Short Review on Emerging Data. Cancer Manag. Res..

[31] Al-Sawaf O., Zhang C., Tandon M., Sinha A., Fink A.-M., Robrecht S., Samoylova O., Liberati A.M., Pinilla-Ibarz J., Opat S. (2020). Venetoclax plus obinutuzumab versus chlorambucil plus obinutuzumab for previously untreated chronic lymphocytic leukaemia (CLL14): Follow-up results from a multicentre, open-label, randomised, phase 3 trial. Lancet Oncol..

[32] Kater A.P., Owen C., Moreno C., Follows G., Munir T., Levin M.-D., Benjamini O., Janssens A., Osterborg A., Robak T. (2022). Fixed-Duration Ibrutinib-Venetoclax in Patients with Chronic Lymphocytic Leukemia and Comorbidities. NEJM Evid..

[33] Tam C.S., Brown J.R., Kahl B.S., Ghia P., Giannopoulos K., Jurczak W., Šimkovič M., Shadman M., Österborg A., Laurenti L. (2022). Zanubrutinib versus bendamustine and rituximab in untreated chronic lymphocytic leukaemia and small lymphocytic lymphoma (SEQUOIA): A randomised, controlled, phase 3 trial. Lancet Oncol..

[34] Simon F., Giza A., Robrecht S., Fink A.-M., Cramer P., von Tresckow J., Fürstenau M., Goede V., Tausch E., Schneider C. (2022). Pooled analysis of first-line treatment with targeted agents in patients with chronic lymphocytic leukemia aged 80 years and older. Leuk. Lymphoma.

[35] Tian Z., Liu M., Fang X., Zhou X., Li P., Li Y., Zhang L., Liu F., Zhang Y., Wang X. (2022). Distinct Age-Related Clinical Features and Risk Assessment in Chinese with Chronic Lymphocytic Leukemia. Front. Oncol..

[36] Richardson R.B. (2013). p53 mutations associated with aging-related rise in cancer incidence rates. Cell Cycle.

[37] Strati P., Parikh S.A., Chaffee K.G., Kay N.E., Call T.G., Achenbach S.J., Cerhan J.R., Slager S.L., Shanafelt T.D. (2017). Relationship between co-morbidities at diagnosis, survival and ultimate cause of death in patients with chronic lymphocytic leukaemia (CLL): A prospective cohort study. Br. J. Haematol..

[38] Nabhan C., Mato A., Flowers C.R., Grinblatt D.L., Lamanna N., Weiss M.A., Davids M.S., Swern A.S., Bhushan S., Sullivan K. (2017). Characterizing and prognosticating chronic lymphocytic leukemia in the elderly: Prospective evaluation on 455 patients treated in the United States. BMC Cancer.

[39] Steingrímsson V., Lund S.H., Dickman P.W., Weibull C.E., Björkholm M., Landgren O., Kristinsson S.Y. (2022). Survival, causes of death, and the prognostic role of comorbidities in chronic lymphocytic leukemia in the pre-ibrutinib era: A population-based study. Eur. J. Haematol..

[40] Rotbain E.C., Niemann C.U., Rostgaard K., da Cunha-Bang C., Hjalgrim H., Frederiksen H. (2021). Mapping comorbidity in chronic lymphocytic leukemia: Impact of individual comorbidities on treatment, mortality, and causes of death. Leukemia.

[41] da Cunha-Bang C., Simonsen J., Rostgaard K., Geisler C., Hjalgrim H., Niemann C.U. (2016). Improved survival for patients diagnosed with chronic lymphocytic leukemia in the era of chemo-immunotherapy: A Danish population-based study of 10455 patients. Blood Cancer J..

[42] Binet J.L., Auquier A., Dighiero G., Chastang C., Piguet H., Goasguen J., Vaugier G., Potron G., Colona P., Oberling F. (1981). A new prognostic classification of chronic lymphocytic leukemia derived from a multivariate survival analysis. Cancer.

[43] Rai K.R., Sawitsky A., Cronkite E.P., Chanana A.D., Levy R.N., Pasternack B.S. (1975). Clinical staging of chronic lymphocytic leukemia. Blood.

[44] Molica S., Shanafelt T.D., Giannarelli D., Gentile M., Mirabelli R., Cutrona G., Levato L., Di Renzo N., Di Raimondo F., Musolino C. (2016). The chronic lymphocytic leukemia international prognostic index predicts time to first treatment in early CLL: Independent validation in a prospective cohort of early stage patients. Am. J. Hematol..

[45] Molica S., Giannarelli D., Levato L., Mirabelli R., Gentile M., Morabito F. (2017). Assessing time to first treatment in early chronic lymphocytic leukemia (CLL): A comparative performance analysis of five prognostic models with inclusion of CLL-international prognostic index (CLL-IPI). Leuk. Lymphoma.

[46] González-Gascón-Y-Marín I., Muñoz-Novas C., Figueroa I., Hernández-Sánchez M., Rodríguez-Vicente A.-E., Quijada-Álamo M., Pérez-Carretero C., Moreno C., Collado R., Espinet B. (2020). Prognosis Assessment of Early-Stage Chronic Lymphocytic Leukemia: Are We Ready to Predict Clinical Evolution Without a Crystal Ball?. Clin. Lymphoma Myeloma Leuk..

[47] Molica S., Giannarelli D., Mirabelli R., Levato L., Shanafelt T.D. (2018). Chronic lymphocytic leukemia international prognostic index (CLL-IPI) in patients receiving chemoimmuno or targeted therapy: A systematic review and meta-analysis. Ann. Hematol..

[48] Goede V., Bahlo J., Kutsch N., Fischer K., Fink A.M., Fingerle-Rowson G., Stilgenbauer S., Bergmann M.A., Eichhorst B.F., Hallek M. (2016). Evaluation of the International Prognostic Index for Chronic Lymphocytic Leukemia (CLL-IPI) in Elderly Patients with Comorbidities: Analysis of the CLL11 Study Population. Blood.

[49] González-Gascón-y-Marín I., Muñoz-Novas C., Rodríguez-Vicente A.-E., Quijada-Álamo M., Hernández-Sánchez M., Pérez-Carretero C., Ramos-Ascanio V., Hernández-Rivas J.-Á. (2021). From Biomarkers to Models in the Changing Landscape of Chronic Lymphocytic Leukemia: Evolve or Become Extinct. Cancers.

[50] Kutsch N. (2021). CLL-IPI: Valid in the era of oral inhibitors?. Blood.

[51] Condoluci A., Terzi di Bergamo L., Langerbeins P., Hoechstetter M.A., Herling C.D., De Paoli L., Delgado J., Rabe K.G., Gentile M., Doubek M. (2020). International prognostic score for asymptomatic early-stage chronic lymphocytic leukemia. Blood.

[52] Wierda W.G., O’Brien S., Wang X., Faderl S., Ferrajoli A., Do K.-A., Garcia-Manero G., Cortes J., Thomas D., Koller C.A. (2011). Multivariable model for time to first treatment in patients with chronic lymphocytic leukemia. J. Clin. Oncol..

[53] Delgado J., Doubek M., Baumann T., Kotaskova J., Molica S., Mozas P., Rivas-Delgado A., Morabito F., Pospisilova S., Montserrat E. (2017). Chronic lymphocytic leukemia: A prognostic model comprising only two biomarkers (IGHV mutational status and FISH cytogenetics) separates patients with different outcome and simplifies the CLL-IPI. Am. J. Hematol..

[54] Hoechstetter M.A., Busch R., Eichhorst B., Bühler A., Winkler D., Bahlo J., Robrecht S., Eckart M.J., Vehling-Kaiser U., Jacobs G. (2020). Prognostic model for newly diagnosed CLL patients in Binet stage A: Results of the multicenter, prospective CLL1 trial of the German CLL study group. Leukemia.

[55] Meiseles A., Paley D., Ziv M., Hadid Y., Rokach L., Tadmor T. (2022). Explainable machine learning for chronic lymphocytic leukemia treatment prediction using only inexpensive tests. Comput. Biol. Med..

[56] Cuturello F., Pozzo F., Villegas Garcia E.N., Rossi F.M., Degan M., Nanni P., Cattarossi I., Zaina E., Varaschin P., Braida A. (2022). An Unsupervised Machine Learning Method Stratifies Chronic Lymphocytic Leukemia Patients in Novel Categories with Different Risk of Early Treatment. Blood.

[57] ePrognosis—About. https://eprognosis.ucsf.edu/about.php.

[58] Mohile S.G., Dale W., Somerfield M.R., Schonberg M.A., Boyd C.M., Burhenn P.S., Canin B., Cohen H.J., Holmes H.M., Hopkins J.O. (2018). Practical Assessment and Management of Vulnerabilities in Older Patients Receiving Chemotherapy: ASCO Guideline for Geriatric Oncology. J. Clin. Oncol..

[59] Koll T.T., Rosko A.E. (2018). Frailty in Hematologic Malignancy. Curr. Hematol. Malig. Rep..

[60] Okoli G.N., Stirling M., Racovitan F., Lam O.L., Reddy V.K., Copstein L., Hsu T., Abou-Setta A.M., Dawe D.E. (2021). Integration of geriatric assessment into clinical oncology practice: A scoping review. Curr. Probl. Cancer.

[61] Klepin H.D. (2019). Ready for prime time: Role for geriatric assessment to improve quality of care in hematology practice. Blood.

[62] Choi J.-Y., Kim K.-I. (2022). Assessing frailty using comprehensive geriatric assessment in older patients with hematologic malignancy. Blood Res..

[63] DuMontier C., Uno H., Hshieh T., Zhou G., Chen R., Magnavita E.S., Mozessohn L., Javedan H., Stone R.M., Soiffer R.J. (2022). Randomized controlled trial of geriatric consultation versus standard care in older adults with hematologic malignancies. Haematologica.

[64] Goede V., Neuendorff N.R., Schulz R.-J., Hormigo A.-I., Martinez-Peromingo F.J., Cordoba R. (2021). Frailty assessment in the care of older people with haematological malignancies. Lancet Healthy Longev..

[65] Cordoba R., Eyre T.A., Klepin H.D., Wildes T.M., Goede V. (2021). A comprehensive approach to therapy of haematological malignancies in older patients. Lancet Haematol..

[66] Goede V., Stauder R. (2019). Multidisciplinary care in the hematology clinic: Implementation of geriatric oncology. J. Geriatr. Oncol..

[67] Williams G.R., Hopkins J.O., Klepin H.D., Lowenstein L.M., Mackenzie A., Mohile S.G., Somerfield M.R., Dale W. (2023). Practical Assessment and Management of Vulnerabilities in Older Patients Receiving Systemic Cancer Therapy: ASCO Guideline Questions and Answers. JCO Oncol. Pr..

[68] Extermann M., Boler I., Reich R.R., Lyman G.H., Brown R.H., DeFelice J., Levine R.M., Lubiner E.T., Reyes P., Schreiber F.J. (2012). Predicting the risk of chemotherapy toxicity in older patients: The Chemotherapy Risk Assessment Scale for High-Age Patients (CRASH) score. Cancer.

[69] Hurria A., Mohile S., Gajra A., Klepin H., Muss H., Chapman A., Feng T., Smith D., Sun C.-L., De Glas N. (2016). Validation of a Prediction Tool for Chemotherapy Toxicity in Older Adults with Cancer. J. Clin. Oncol..

[70] Johnson P.C., Woyach J.A., Ulrich A., Marcotte V., Nipp R.D., Lage D.E., Nelson A.M., Newcomb R.A., Rice J., Lavoie M.W. (2023). Geriatric assessment measures are predictive of outcomes in chronic lymphocytic leukemia. J. Geriatr. Oncol..

[71] Gordon M.J., Churnetski M., Alqahtani H., Rivera X., Kittai A., Amrock S.M., James S., Hoff S., Manda S., Spurgeon S.E. (2018). Comorbidities predict inferior outcomes in chronic lymphocytic leukemia treated with ibrutinib. Cancer.

[72] Moreno C., Greil R., Demirkan F., Tedeschi A., Anz B., Larratt L., Simkovic M., Novak J., Strugov V., Gill D. (2022). First-line treatment of chronic lymphocytic leukemia with ibrutinib plus obinutuzumab versus chlorambucil plus obinutuzumab: Final analysis of the randomized, phase III iLLUMINATE trial. Haematologica.

[73] Rotbain E.C., Gordon M.J., Vainer N., Frederiksen H., Hjalgrim H., Danilov A.V., Niemann C.U. (2022). The CLL comorbidity index in a population-based cohort: A tool for clinical care and research. Blood Adv..

[74] Gordon M.J., Kaempf A., Sitlinger A., Shouse G., Mei M., Brander D.M., Salous T., Hill B.T., Alqahtani H., Choi M. (2021). The Chronic Lymphocytic Leukemia Comorbidity Index (CLL-CI): A Three-Factor Comorbidity Model. Clin. Cancer Res..

[75] Lyon A.R., López-Fernández T., Couch L.S., Asteggiano R., Aznar M.C., Bergler-Klein J., Boriani G., Cardinale D., Cordoba R., Cosyns B. (2022). 2022 ESC Guidelines on cardio-oncology developed in collaboration with the European Hematology Association (EHA), the European Society for Therapeutic Radiology and Oncology (ESTRO) and the International Cardio-Oncology Society (IC-OS). Eur. Heart J..

[76] Sestier M., Hillis C., Fraser G., Leong D. (2021). Bruton’s tyrosine kinase Inhibitors and Cardiotoxicity: More Than Just Atrial Fibrillation. Curr. Oncol. Rep..

[77] Lipsky A., Lamanna N. (2020). Managing toxicities of Bruton tyrosine kinase inhibitors. Hematology.

[78] Gale R.P., Chapel H.M., Bunch C., Rai K.R., Foon K., Courter S.G., Tait D., Cooperative Group for the Study of Immunoglobulin in Chronic Lymphocytic Leukemia (1988). Intravenous immunoglobulin for the prevention of infection in chronic lymphocytic leukemia. A randomized, controlled clinical trial. N. Engl. J. Med..

[79] Wierda W.G., Tambaro F.P. (2020). How I manage CLL with venetoclax-based treatments. Blood.

[80] Bonanad S., De la Rubia J., Gironella M., Pérez Persona E., González B., Fernández Lago C., Arnan M., Zudaire M., Hernández Rivas J.A., Soler A. (2015). Development and psychometric validation of a brief comprehensive health status assessment scale in older patients with hematological malignancies: The GAH Scale. J. Geriatr. Oncol..

[81] Cruz-Jentoft A.J., González B., de la Rubia J., Hernández Rivas J.Á., Soler J.A., Fernández Lago C., Arnao M., Gironella M., Pérez Persona E., Zudaire M.T. (2017). Further psychometric validation of the GAH scale: Responsiveness and effect size. J. Geriatr. Oncol..

[82] de la Rubia J., González B., Cruz-Jentoft A.J., Iglesias L., Jarque I., Persona E.P., Lluch R., Marrero C., Zudaire M., Gironella M. (2023). Geriatric assessment in hematology scale predicts treatment tolerability in older patients diagnosed with hematological malignancies: The RETROGAH study. J. Geriatr. Oncol..

[83] Chapman A.E., Elias R., Plotkin E., Lowenstein L.M., Swartz K. (2021). Models of Care in Geriatric Oncology. J. Clin. Oncol..

[84] Loewenthal J., DuMontier C., Cooper L., Frain L., Waldman L.S., Streiter S., Cardin K., Tulebaev S., Javedan H., Orkaby A.R. (2020). Adaptation of the comprehensive geriatric assessment to a virtual delivery format. Age Ageing.

[85] Wildiers H., Heeren P., Puts M., Topinkova E., Janssen-Heijnen M.L.G., Extermann M., Falandry C., Artz A., Brain E., Colloca G. (2014). International Society of Geriatric Oncology consensus on geriatric assessment in older patients with cancer. J. Clin. Oncol..

[86] Loh K.P., Soto-Perez-de-Celis E., Hsu T., de Glas N.A., Battisti N.M.L., Baldini C., Rodrigues M., Lichtman S.M., Wildiers H. (2018). What Every Oncologist Should Know About Geriatric Assessment for Older Patients with Cancer: Young International Society of Geriatric Oncology Position Paper. J. Oncol. Pract..

[87] Wall S.A., Huang Y., Keiter A., Funderburg A., Kloock C., Yuhasz N., Gure T.R., Folefac E., Stevens E., Presley C.J. (2021). Integration of a Geriatric Assessment with Intervention in the Care of Older Adults with Hematologic Malignancies. Front. Oncol..

[88] Garric M., Sourdet S., Cabarrou B., Steinmeyer Z., Gauthier M., Ysebaert L., Beyne-Rauzy O., Gerard S., Lozano S., Brechemier D. (2021). Impact of a comprehensive geriatric assessment on decision-making in older patients with hematological malignancies. Eur. J. Haematol..

[89] Liu M.A., DuMontier C., Murillo A., Hshieh T.T., Bean J.F., Soiffer R.J., Stone R.M., Abel G.A., Driver J.A. (2019). Gait speed, grip strength, and clinical outcomes in older patients with hematologic malignancies. Blood.

[90] Soo W.-K., King M., Pope A., Parente P., Darzins P., Davis I.D. (2020). Integrated geriatric assessment and treatment (INTEGERATE) in older people with cancer planned for systemic anticancer therapy. J. Clin. Oncol..

[91] Goede V., Bahlo J., Chataline V., Eichhorst B., Dürig J., Stilgenbauer S., Kolb G., Honecker F., Wedding U., Hallek M. (2016). Evaluation of geriatric assessment in patients with chronic lymphocytic leukemia: Results of the CLL9 trial of the German CLL study group. Leuk. Lymphoma.

[92] Molica S., Giannarelli D., Levato L., Mirabelli R., Levato D., Lentini M., Piro E. (2019). A simple score based on geriatric assessment predicts survival in elderly newly diagnosed chronic lymphocytic leukemia patients. Leuk. Lymphoma.

[93] Van Der Straten L., Stege C.A.M., Kersting S., Nasserinejad K., Dubois J., Dobber J.A., Mellink C.H.M., van der Kevie-Kersemaekers A.-M.F., Evers L.M., de Boer F. (2023). Fixed-duration venetoclax plus obinutuzumab improves quality of life and geriatric impairments in FCR-unfit CLL patients. Blood.

[94] Hallek M., Fischer K., Fingerle-Rowson G., Fink A.M., Busch R., Mayer J., Hensel M., Hopfinger G., Hess G., von Grünhagen U. (2010). Addition of rituximab to fludarabine and cyclophosphamide in patients with chronic lymphocytic leukaemia: A randomised, open-label, phase 3 trial. Lancet.

[95] Eichhorst B., Fink A.-M., Bahlo J., Busch R., Kovacs G., Maurer C., Lange E., Köppler H., Kiehl M., Sökler M. (2016). First-line chemoimmunotherapy with bendamustine and rituximab versus fludarabine, cyclophosphamide, and rituximab in patients with advanced chronic lymphocytic leukaemia (CLL10): An international, open-label, randomised, phase 3, non-inferiority trial. Lancet Oncol..

[96] Goede V., Fischer K., Busch R., Engelke A., Eichhorst B., Wendtner C.M., Chagorova T., de la Serna J., Dilhuydy M.-S., Illmer T. (2014). Obinutuzumab plus chlorambucil in patients with CLL and coexisting conditions. N. Engl. J. Med..

[97] Barr P.M., Owen C., Robak T., Tedeschi A., Bairey O., Burger J.A., Hillmen P., Coutre S.E., Dearden C., Grosicki S. (2022). Up to 8-year follow-up from RESONATE-2: First-line ibrutinib treatment for patients with chronic lymphocytic leukemia. Blood Adv..

[98] Woyach J.A., Ruppert A.S., Heerema N.A., Zhao W., Booth A.M., Ding W., Bartlett N.L., Brander D.M., Barr P.M., Rogers K. (2021). Long-Term Results of Alliance A041202 Show Continued Advantage of Ibrutinib-Based Regimens Compared with Bendamustine Plus Rituximab (BR) Chemoimmunotherapy. Blood.

[99] Sharman J.P., Egyed M., Jurczak W., Skarbnik A., Pagel J.M., Flinn I.W., Kamdar M., Munir T., Walewska R., Corbett G. (2022). Efficacy and safety in a 4-year follow-up of the ELEVATE-TN study comparing acalabrutinib with or without obinutuzumab versus obinutuzumab plus chlorambucil in treatment-naïve chronic lymphocytic leukemia. Leukemia.

[100] Byrd J.C., Hillmen P., Ghia P., Kater A.P., Chanan-Khan A., Furman R.R., O’Brien S., Yenerel M.N., Illés A., Kay N. (2021). Acalabrutinib Versus Ibrutinib in Previously Treated Chronic Lymphocytic Leukemia: Results of the First Randomized Phase III Trial. J. Clin. Oncol..

[101] Brown J.R., Eichhorst B., Hillmen P., Jurczak W., Kaźmierczak M., Lamanna N., O’Brien S.M., Tam C.S., Qiu L., Zhou K. (2023). Zanubrutinib or Ibrutinib in Relapsed or Refractory Chronic Lymphocytic Leukemia. N. Engl. J. Med..

[102] Mato A.R., Thompson M., Allan J.N., Brander D.M., Pagel J.M., Ujjani C.S., Hill B.T., Lamanna N., Lansigan F., Jacobs R. (2018). Real-world outcomes and management strategies for venetoclax-treated chronic lymphocytic leukemia patients in the United States. Haematologica.

[103] Mato A.R., Nabhan C., Thompson M.C., Lamanna N., Brander D.M., Hill B., Howlett C., Skarbnik A., Cheson B.D., Zent C. (2018). Toxicities and outcomes of 616 ibrutinib-treated patients in the United States: A real-world analysis. Haematologica.

[104] Ysebaert L., Aurran-Schleinitz T., Dartigeas C., Dilhuydy M.-S., Feugier P., Michallet A.-S., Tournilhac O., Dupuis J., Sinet P., Albrecht C. (2017). Real-world results of ibrutinib in relapsed/refractory CLL in France: Early results on a large series of 428 patients. Am. J. Hematol..

[105] Chronische Lymphatische Leukämie (CLL). https://www.onkopedia.com/de/onkopedia/guidelines/chronische-lymphatische-leukaemie-cll.

[106] Guía de Tratamiento de la LLC. https://www.gellc.es/noticias/110-guia-de-tratamiento-de-la-llc.

[107] Zuccarino S., Monacelli F., Antognoli R., Nencioni A., Monzani F., Ferrè F., Seghieri C., Antonelli Incalzi R. (2022). Exploring Cost-Effectiveness of the Comprehensive Geriatric Assessment in Geriatric Oncology: A Narrative Review. Cancers.

